# Influence of Filler Content and Polishing on *Candida* and *Streptococci* Biofilm Formation in Resin-Based Composites: An In Vitro Evaluation

**DOI:** 10.1155/cjid/5734405

**Published:** 2025-06-22

**Authors:** Zahra Zareshahrabadi, Sarina Sahmeddini, Marzieh Meimandinezhad, Afsoon Tondari, Kamiar Zomorodian

**Affiliations:** ^1^Basic Sciences in Infectious Diseases Research Center, Shiraz University of Medical Sciences, Shiraz, Iran; ^2^Arthur A. Dugoni School of Dentistry, University of the Pacific, San Francisco, California, USA; ^3^Dentistry School, Islamic Azad University of Shiraz, Shiraz, Iran; ^4^Department of Restorative Dentistry, Dental Branch, Shiraz Azad University, Shiraz, Iran; ^5^Department of Medical Parasitology and Mycology, Shiraz University of Medical Sciences, Shiraz, Iran

**Keywords:** biofilm metabolic activity, *Candida*, resin-based composites, *Streptococcus*

## Abstract

**Aim:** The purpose of this study was to investigate the impact of filler content and polishing of resin-based composites on in vitro biofilm formation of *Candida* and *Streptococci* species.

**Materials and Methods:** Specimens of four commercially available resin-based composites including Z100, P60, Z250, and Z350, with different filler amounts and volumes, were prepared according to the manufacturer's instructions. Each group was divided into polished and unpolished specimens, which were then placed in a 24-well tissue culture plate with microbial suspension and incubated. The XTT technique was used to evaluate biofilm formation.

**Results:** Z250 resin-based composites, which had the highest percentage of filler (68%), had the highest biofilm metabolic activity. A significantly less microbial biofilm metabolic activity was noted on P60 polished resin-based composites than on unpolished groups (*p* < 0.001). Polishing procedures reduce biofilm metabolic activity. *Streptococcus salivarius* produced the least biofilm metabolic activity among the *Streptococcal* species (*p* < 0.001). However, there were no statistically significant differences between *Candida* species in the biofilm metabolic activity.

**Conclusion:** The results revealed that the amount of filler in resin-based composites had a major impact on the biofilm metabolic activity. Therefore, resin-based composites with a minimized excess resin matrix, minimized filler amount, and smoother surfaces might be more useful in reducing biofilm metabolic activity and secondary caries. These findings may be useful for modifying novel resin-based composite formulations to improve oral health and patient wellbeing.

## 1. Introduction

Resin-based composites (RBCs) represent the cornerstone of direct restorative materials in contemporary dental practices, enjoying widespread adoption due to their versatility, esthetic, and compatibility with the principles of minimally invasive dentistry [1–3]. Despite their pervasive use, concerns regarding their durability and the heightened incidence of secondary caries have emerged as significant challenges, precipitating clinical failures [4]. Literature suggests that failures within the initial 5 years of application are predominantly attributed to the inappropriate selection of RBCs and suboptimal application techniques; beyond this period, failure rates approach 40%, primarily due to secondary caries [5]. Consequently, a substantial portion of dental practice resources is allocated toward the replacement of these restorations, underlining a critical area for improvement in dental materials science and operative techniques. The maintenance of a harmonious equilibrium between the host and the microbial flora within the oral environment is essential for the preservation of health in both native and restored dental structures. This necessity stems from the fact that both biological and artificial surfaces present within the oral cavity are invariably enveloped by microbial biofilms. These biofilms are complex assemblies of microbial entities embedded within a self-produced matrix of extracellular polymeric substances. Encased within this protective matrix, microbial communities are afforded a degree of resilience against a range of environmental stressors, including ultraviolet radiation, desiccation, and host immunological defenses. Additionally, the conditions prevalent within the oral microbiome promote the proliferation and settlement of diverse microorganisms, encompassing both fungal and bacterial species. This process culminates in the establishment of biofilms on the surfaces of restorative materials, an event that significantly contributes to the etiology of secondary caries, thereby underscoring the intricate interplay between microbial biofilms and dental health [6, 7].

*Streptococci* and *Candida* represent the predominant microbial genera within the biofilms associated with secondary carious lesions [8, 9]. Notably, *Candida dubliniensis* and *Candida albicans* have been identified within populations exhibiting active carious processes [10]. These findings underscore the significant role these microbial species play in the pathogenesis of dental caries, highlighting their potential as targets for therapeutic interventions aimed at mitigating the progression of such lesions [11]. The virulence of these microorganisms is related to their high adhesion capability to teeth, oral mucosa, and restorative materials, especially RBCs [12, 13].

Many manufacturers have produced a broad range of resin composites with different material characteristics, including surface roughness, hydrophobicity, surface energy, material composition, matrix type, filler size and volume, and filler configuration. The variability in these material characteristics directly impacts the interaction between the resin composites and the microbial community, potentially affecting the composite's resistance to biofilm formation and, by extension, its long-term performance and durability in dental applications [14–16]. The design of RBCs with unfavorable conditions for microbial adhesion and colonization is a promising approach in current dental materials science and can reduce the chances of secondary caries and oral inflammatory diseases, such as stomatitis and chronic periodontitis. The findings of this study have important clinical implications for the selection and use of RBCs in dental restorations. The formation of microbial biofilms on RBCs is a major factor contributing to the failure of restorations, particularly through the development of secondary caries. Hence, evaluating microbial biofilms on RBCs with different characteristics is important for achieving long-term success in oral healing and protection from oral diseases. The objective of this study was to determine the relationship among filler content, surface roughness, and biofilm formation of *Streptococcus* and *Candida* species.

## 2. Materials and Methods

### 2.1. RBC Specimen Preparation

Four commercially available RBCs (3 M ESPE, St. Paul, MN, USA), including Z100, P60, Z250, and Z350, were used in this study (Table 1) [17, 18]. Custom-made cylindrical molds (8-mm diameter and 2-mm height) with circular holes were used to prepare 200 RBC discs. The RBCs used in this study (Z100, P60, Z250, and Z350) vary in filler volume and composition. While the specific details of the resin matrix and silane coupling agents are proprietary and not disclosed by the manufacturers, the filler content and surface characteristics were the primary focus of this investigation.

For this study, 50 discs were made of each RBC material. The material was poured into the mold, and both sides were covered with a transparent strip and glass slides under constant pressure before being light-cured (iLed Woodpecker, 2300 mw/cm^2^) for 40 s. All RBCs were handled in strict compliance with their manufacturers' instructions. The samples were removed, and half of each group's specimens were mechanically polished with coarse (100 microns), medium (60 microns), fine (40 microns), and superfine (20 microns) aluminum oxide abrasive discs (3 M Sof-Lex series). The other half remained untouched. Polishing was conducted for a standardized duration of 30 s per disc, starting with the coarsest to the finest. This sequential polishing was applied to ensure a uniform and gradual reduction of surface roughness. The endpoint of polishing was determined visually under a magnification of 10x and confirmed by achieving a consistent reflective gloss, which indicates a smooth surface free of any visible scratches under the specified magnification. In summary, a total of 200 RBC discs were prepared, corresponding to each of the four types of resin, allocating 50 discs per RBC type. These 50 RBC discs of each composite type were then distributed among seven organisms under study, allocating 6 discs to each species. Of these six discs, three were polished and three were unpolished. The remaining eight discs of each resin type were reserved for control purposes or potential additional requirements.

### 2.2. Disinfection of Specimens

Following their preparation, all the specimens were stored in a flask containing 90% ethanol for 1 min, washed in running distilled water, and then in a dry environment at room temperature and exposed to ultraviolet light for 20 min to kill any remaining microorganisms. While ethanol disinfection can remove unpolymerized monomers from the surface of RBCs and may be regarded as a mild form of artificial aging, the short exposure time (1 min) was chosen to minimize any significant alterations to the surface properties. Future studies should include characterization methods to confirm that the disinfection process does not significantly affect surface roughness or other surface characteristics.

### 2.3. Microbial Strains

For the investigation of biofilm metabolic activity, this study employed a selection of bacterial and fungal species known for their relevance in oral biofilms. The bacterial strains included *S. mutans* (ATCC 35668), *S. sobrinus* (ATCC 27607), and *S. salivarius* (ATCC 9222). The fungal species utilized were *C. albicans* (ATCC 35668) and *C. dubliniensis* (CBS 8501). Additionally, the study also incorporated two clinical isolates, one of *C. albicans* and another of *S. mutans* were previously [19–21] detected in plaque-biofilm and identified by conventional and molecular methods, to assess their biofilm-forming capabilities in comparison with the ATCC strains. This diverse microbial panel was chosen to provide a comprehensive understanding of the biofilm metabolic activity on RBCs, reflecting a range of organisms implicated in dental caries and oral health complications. The bacterial strains were stored at −80°C in 20% glycerol to preserve viability. Fungal strains were maintained on sabouraud dextrose agar (SDA, HiMedia, India) slants and stored at 4°C for short-term use, with periodic subculturing every 4–6 weeks to ensure freshness [22, 23].

### 2.4. Assays for Measuring of Microbial Biofilm Metabolic Activity

#### 2.4.1. Preparing and Growth of Biofilms

To initiate biofilm metabolic activity assays, standard strains of *Streptococcus* species were cultivated in brain heart infusion (BHI, Merck, Germany) medium and incubated for 24 h at 37°C. *Candida* species were cultivated in SDA and incubated for 24 h at 32°C. Post incubation, one of the colony loops was transferred to 20 mL of broth media in 250-mL Erlenmeyer flasks and incubated in an orbital shaker (100 rpm) at 30°C overnight in an aerobic environment. Microbial cells were harvested by centrifugation at a specific speed and time appropriate for the cell type under study. After cultivation under the designated conditions, the culture medium containing the cells was transferred to sterile centrifuge tubes. The tubes were then centrifuged at a speed of 4000 rpm for 10 min at 4°C to pellet the cells. This centrifugation speed and time were optimized based on preliminary experiments to ensure efficient pelleting of both *Streptococcus* and *Candida* species, which typically have different cell densities and sedimentation characteristics. Specifically, *Streptococcus* species, being Gram-positive cocci, tend to form aggregates, requiring careful handling to avoid disruption of the pellet during resuspension. Conversely, *Candida* species, which are yeast-like fungi, may require a gentler approach to maintain cell viability and integrity [24, 25]. The supernatant was carefully decanted, and the cell pellet was retained for subsequent washing and experimental procedures. Microbial cells were washed twice in sterile phosphate-buffered saline (PBS) (0.8% [w/v], NaCl (Merck); 0.02% [w/v], KH2PO4 (Merck); 0.31% [w/v], Na2HPO4+12 H2O (Merck), 0.02% [w/v], KCl (Panreac); pH 7.4). The *Streptococcus* and *Candida* strains were then resuspended in Mueller Hinton broth (MHB) and RPMI 1640, respectively. Microbial suspensions were adjusted to 0.5 McFarland standard turbidity using the spectrophotometric method that is a stock suspension of 1–5 × 10^6^ cells/mL (after counting with a hemocytometer, cell densities were altered to 1.0 × 10^6^ cells/mL) for *Candida* species and 1–1.5 × 10^8^ cells/mL (0.08–0.1 absorbance) for bacterial species. The resulting *Streptococcus* and *Candida* suspensions (500 μL) were added to each well of a 24-well tissue culture plate (Corning, St. Louis, MO, USA). Louis, MO, USA) containing RBC samples. For our experimental setup, we employed both positive and negative controls: media containing *Candida* and *Streptococcus* strains but lacking RBCs were used as positive controls. These wells allow us to assess the maximum metabolic activity of the microbial strains in the absence of any material interference. Media containing only uninoculated broth (without any microbial strains) were used as negative controls. This control helps establish the baseline absorbance readings due to the broth alone, ensuring that any absorbance measured in the test wells is attributable to microbial metabolic activity. The plates were incubated for 48 h at 37°C [26, 27]. All tests were performed in triplicates. The RBC samples without any manipulation in wells containing media and organism were used as positive control.

#### 2.4.2. Quantitative Biofilm Inhibition Measurement

A colorimetric assay was used to calculate the metabolic activity of microbial biofilms. As an indicator of biofilm metabolic activity, a 2,3-bis (2-methoxy-4-nitro-5-sulfo-phenyl)-2H-tetrazolium-5-carbox-anilide (XTT) reduction assay (Sigma, St. Louis, MO, USA) was performed in Ringers lactate (0.5 mg/mL). After filter-sterilized (0.22-μm pore size), the solution was kept at −70°C. The XTT stock solution was combined with menadione sodium bisulfite (10 mM, Sigma-Aldrich, St. Louis, MO, USA) before each assay [28–30]. After 48 h of incubation, the RBC samples were transferred to a new tissue culture plate and washed twice with sterile PBS. Next, a 500-μL aliquot of XTT/menadione (at a volume ratio of 20:1) was added to each well of the 24-well plates. The plates were then incubated at 37°C for 3 h. Finally, the colorimetric alterations at 570 nm using a microplate reader (BMG Labtech, Berlin, Germany) were quantified to assess the changes in sample absorbance (absorbance values are displayed as OD), indicating the degree of biofilm metabolic activity associated with the experimental conditions [28, 31, 32]. Every test was performed in triplicate, and the percentage of metabolic activity was computed as follows:

(1) $$\begin{array}{c} \text{Metabolic activity percentage}=\text{absorbance of control well}\left(+\right)-\frac{\text{absorbance of test well}}{\text{absorbance of control well}\left(+\right)}\times 100. \end{array}$$

#### 2.4.3. Qualitative Observation of the *C. albicans* Biofilm

Scanning electron microscopy (SEM) was used to observe the biofilm that had formed on the surfaces. To examine the ultrastructural nature of grown biofilms and the morphological features of *C. albicans*, RBC samples were fixed in 2.5% glutaraldehyde in 0.1 M phosphate buffer (pH 7.2) at 4°C for 1 h. After being washed in buffer, the samples were postfixed in 1% osmium tetroxide in the same buffer for 30 min. The samples were dehydrated in the graded concentrations of ethanol and critical point dried in CO_2_ (Polaron Critical Point Dryer). They were then coated with colloidal gold (Balzers SCD 050 Sputter Coater, Baltic, Liechtenstein) and viewed under a Leo 435 VP SEM (Oxford Instruments, Oxford, UK) at 15 kV [33].

### 2.5. Data Statistical Analysis

For the statistical analysis in this study, we utilized SPSS version 22 and quantitative data are presented as the mean ± standard deviation (SD). To compare the biofilm metabolic activity of the microbial strains between these types of RBC specimens, a two-way ANOVA test (Duncan's post hoc) was used. Statistical significance was set at *p* ≤ 0.05.

## 3. Results

During the XTT assay, a thin layer of biofilm was observed on the surfaces of all tested discs. Nevertheless, the biofilm metabolic activity of tested microorganisms varied among the materials. We used two-way ANOVA, and the results are summarized in Tables 2, 3, 4, and 5. The results indicated that P60 samples had the lowest quantity of biofilm metabolic activity in comparison with other types of RBCs, as evidenced by a lower absorbance reading compared to the control (*p*-value < 0.001). However, the Z250 samples had the highest biofilm metabolic activity. In addition, all polished discs had a lower biofilm metabolic activity than unpolished discs. The study found significant differences in biofilm metabolic activity across different types of RBCs, with a notable interaction effect observed when considering the polishing treatment (*p*-value < 0.017).

**Figure 1:**
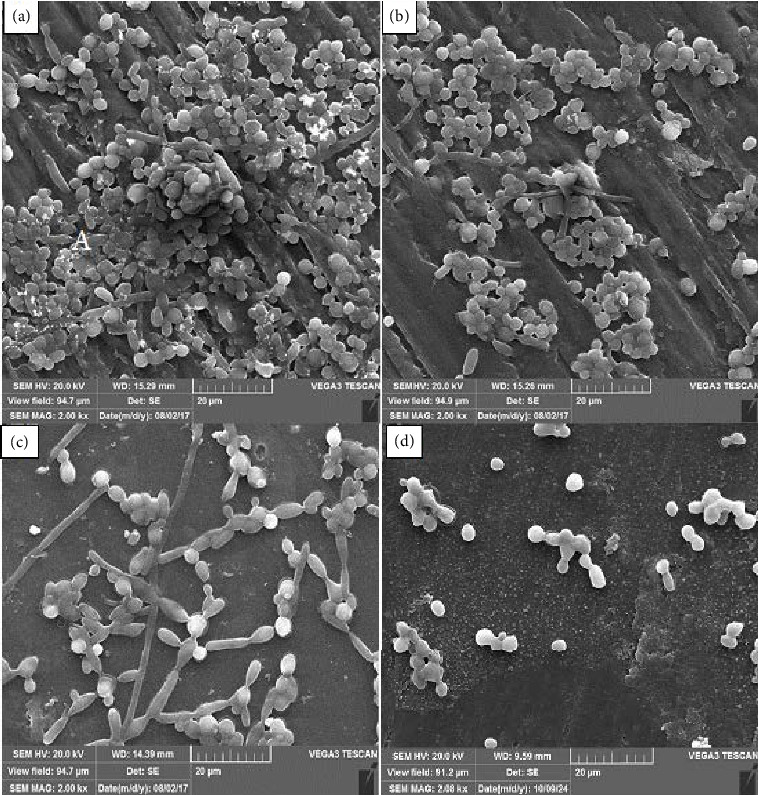
SEM images (×2000 magnification) of *C. albicans* biofilms on polished tested RBC types: (a) Z250; (b) Z100; (c) Z350; (d) P60.

The *Candida* species biofilm metabolic activity by the XTT reduction assay in the four groups is demonstrated in Figure 1. *C. albicans* biofilm metabolic activity was lower than that of *C. dubliniensis*, but the difference was not statistically significant (*p*-value > 0.05). The analysis revealed statistically significant differences in biofilm metabolic activity by *Streptococcus* species across the different RBC groups tested. Notably, *S. salivarius* exhibited the lowest biofilm metabolic activity among the *Streptococcus* species, with this difference being statistically significant (*p*-value < 0.001).

This finding underscores the differential ability of *Streptococcus* species to form biofilms on RBC surfaces, highlighting the potential for targeted strategies in reducing biofilm-associated risks in dental restorations. In the evaluation of the effects of polishing on biofilm metabolic activity by *Candida* and *Streptococcus* species on RBCs, significant findings were observed. Specifically, the unpolished surfaces showed an increase in the biofilm metabolic activity compared to polished surfaces, with mean biofilm densities of 1.75 ± 0.30 and 1.20 ± 0.25, respectively (*p*-value < 0.05). Furthermore, the type of RBCs also played a critical role, with composite P60 exhibiting a lower biofilm metabolic activity compared to composite Z350, indicating a significant material effect (*p*-value < 0.01). The interaction between the type of composite and the polishing treatment was also significant (*p*-value < 0.05).

**Figure 2:**
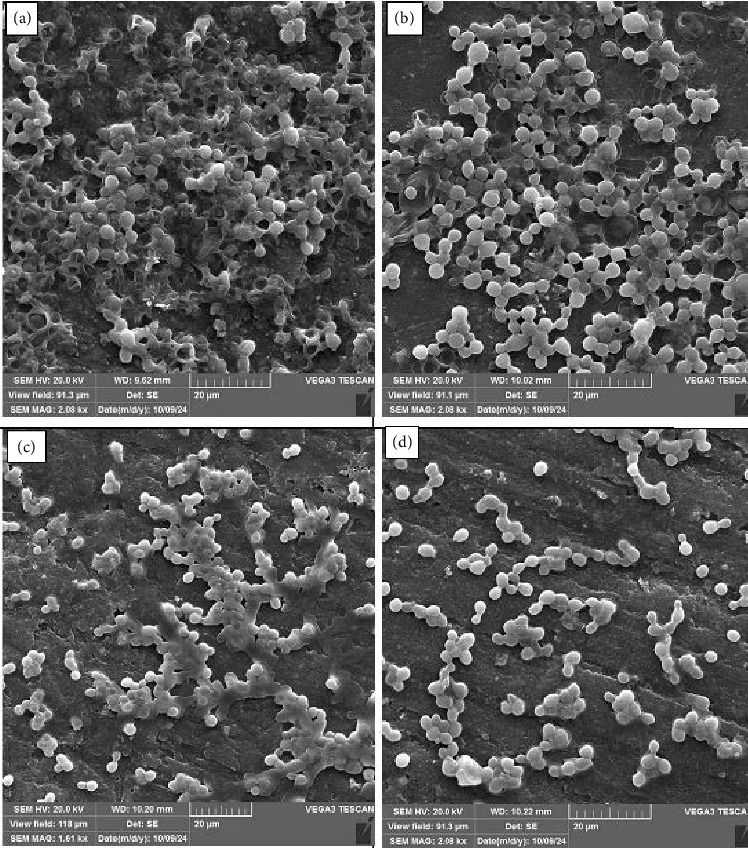
SEM images (×2000 magnification) of *C. albicans* biofilms on unpolished tested RBC types: (a) Z250; (b) Z100; (c) Z350; (d) P60.

SEM was employed to evaluate the morphological changes and biofilm formation of *C. albicans* on different types of polished and unpolished RBCs. The SEM images at 2000 x magnification, as shown in Figures 1 and 2, clearly illustrate the biofilm's structural composition across four types of RBCs. The images reveal a significant variance in biofilm density and structure, with the P60 samples showing considerably less biofilm coverage compared to the Z250, Z350, and Z100 samples. The detailed morphology captured by the SEM highlights the smooth surface of the P60 RBC, which appears to inhibit *C. albicans* adhesion and biofilm formation effectively. This observation underscores the importance of surface polishing in reducing microbial colonization on RBCs; the biofilm architecture was seriously damaged in the P60 polished RBC, and few *C. albicans* cells were observed on these acrylic resins. The smoother surface of the polished P60 reduces the available niches for microbial attachment, which is a critical factor in the reduced biofilm formation observed. The SEM analysis provides a visual confirmation of how surface modifications, such as polishing, can significantly impact the microbial biofilm dynamics on dental materials. The SEM results, which showed distinct differences in biofilm formation across the RBC types, are consistent with the findings from the XTT assay. The XTT assay, a colorimetric method for assessing cellular metabolic activity, indicated a lower metabolic activity in biofilms on the P60 samples compared to those on Z250, Z350, and Z100. This correlation supports the hypothesis that smoother surfaces, as observed in the P60 samples, inhibit biofilm vitality by reducing microbial adhesion and growth.

## 4. Discussion

The scientific literature on dental restorative materials has rigorously examined the physical and chemical characteristics of commonly employed products, including P60, Z100, Z250, and Z350. Notably, P60 and Z250 have been extensively studied due to their remarkable blend of properties such as microhardness, biocompatibility, and color stability. Such investigations are pivotal in ascertaining their adaptability and effectiveness for diverse clinical uses. The detailed analysis of these attributes facilitates informed decision-making in material selection, aiming to improve the performance and durability of dental restorations in a clinical setting [34]. The Z100 RBC has been investigated for its mechanical properties, polymerization shrinkage, and color stability, making it a popular choice for anterior and posterior restorations, and orthodontic attachments [35]. Z350 has also garnered attention in the literature for their enhanced esthetics, high polishability, and improved mechanical characteristics, which contribute to their widespread use in restorative dentistry [36]. By analyzing the scientific literature on these materials, dental professionals can make informed decisions when selecting the most appropriate restorative material for each clinical situation, ensuring optimal patient outcomes. In the current study, according to the different mechanical and physical properties of these restorative materials, in our investigation, the emphasis was placed on examining the propensity for microbial attachment and subsequent biofilm formation on the surfaces of RBCs through an in vitro study. Nevertheless, a lower biofilm formation could also be important in the esthetic area where specific finishing procedures can be applied [37]. This research aimed to elucidate the relationship between the susceptibility of four RBCs with varying surface characteristics and filler contents to the adhesion of *Candida* and *Streptococcus* species. To date, the most commonly used tetrazolium salt among colorimetric assays for the antimicrobial susceptibility testing of bacteria and fungi is XTT, which after reduction yields a water-soluble formazan derivative that can be easily quantified colorimetrically [30]. Despite its popularity, XTT is expensive and problems regarding intra- and interspecies variabilities have been reported [38–40]. The XTT assay measures the metabolic activity of microbial biofilms, which is not necessarily directly proportional to the total biomass or number of cells in the biofilm [40]. While the XTT assay is a widely used method for assessing biofilm metabolic activity, it is important to acknowledge the potential for interspecies variability in the results. Differences in metabolic rates, enzyme activity, and biofilm architecture between *Candida* and *Streptococcus* species can influence the reduction of XTT to formazan, complicating direct comparisons of biofilm metabolic activity. To mitigate this variability, we standardized the experimental conditions for all microbial species, including microbial suspension concentration, incubation time, and XTT incubation period. The findings suggest a direct correlation between the volume of filler in Z100 and Z 250 RBCs and the extent of biofilm metabolic activity; notably, an increase in filler volume and the presence of inorganic particles were associated with heightened biofilm development. In the present study recognized that the biofilm activity observed in P60 and Z350 does not follow a direct correlation with filler content. Despite the higher amount of filler volume in the P60 in comparison with that of the Z350, the P60 composite had less biofilm metabolic activity than the Z350. Therefore, we concluded that biofilm formation on RBC surfaces is complicated and multifactorial. Other factors that can easily affect biofilm formation on RBCs include the material's surface chemical composition, such as filler size, shape, and distribution, as well as matrix composition [41]. According to Choudhary et al.'s study, the Z350 composite has more space between the filler particles than the P60 composite [42]. It can be concluded that in the P60 composite, due to the less space between the filler particles leading to a decrease in the porosity and the excess resin matrix, despite the higher filler volume, less biofilm will be formed. To our knowledge, the specific impact of filler volume on biofilm formation has been sparingly addressed in the existing literature, with the majority of studies focusing on aspects such as filler fraction, size, or weight. This gap highlights the novelty of our investigation, suggesting that understanding the influence of filler volume could provide new insights into optimizing RBC formulations for reduced biofilm formation and improved clinical outcomes. The SEM images revealed a significant variance in biofilm density across the RBCs. The Z250 samples showed the highest biofilm formation, followed by Z100, Z350, and finally P60, which revealed the least biofilm formation. This trend supports with the variances in surface characteristics and filler contents in the RBCs. The Z250, with the highest filler content (68%), provided more niches for microbial adhesion, resulting in the most substantial biofilm formation. Z350, despite its smoother surface, still showed considerable biofilm coverage, likely due to its nanofilled composition and intermediate filler content (55.6%). Z100, with a lower filler content (66%) compared to Z250 (68%), showed more biofilm formation than Z350 and P60. The P60, with its smoother surface and improved filler distribution, demonstrated the least biofilm formation, highlighting the importance of surface smoothness in reducing microbial adhesion. It is important to note that the matrix composition of the composites used in this study varies, with Z100 containing TEGDMA, while P60 and Z250 do not. Additionally, although P60 and Z250 share the same resin components (Bis-GMA, UDMA, and Bis-EMA), their ratios may differ. While this study focused on the influence of filler content and surface roughness on biofilm formation, it is acknowledged that the properties of RBCs are influenced by multiple factors, including matrix composition, filler size, distribution, and silane coupling agents. For example, the differences in flexural strength between Z100 (114 MPa) and Z250 (138.2 MPa), despite their similar filler contents, underscore the multifactorial nature of composite properties. These variations can be attributed to differences in matrix composition, filler characteristics, and other factors. These differences in matrix composition could influence the material's hydrophobicity, surface energy, and susceptibility to microbial adhesion. Ionescu et al. concluded that RBCs with nanoscaled filler particles, such as Z350, have the lowest biofilm formation. However, in the multispecies model, which is more similar to the mouth, a huge effect of the resin matrix blend was observed compared to filler particles [14]. A similar study found that the ratio of resin matrix and filler particles on the surface of RBCs has a great impact on the formation of biofilm and that reducing resin matrix exposure may be help reduce the biofilm formation on the surface of RBCs [43]. Pereira et al., on the other hand, showed that a nanofilled RBC (Filtek Z350™) formed the least biofilm when compared to nanohybrid, microhybrid (P60), and bulk-filled RBCs. They declared that nanosized inorganic fillers could have broader particle distribution and smoother composite surfaces following the same finishing and polishing processes, resulting in the reduction of microorganism adhesion [44]. Surfaces with low surface roughness have poor wetting by saliva and greater resistance to biofilm formation [45]. Soleiman et al. also declared that regardless of the restorative material, the smoothest surfaces have the lowest adhesion of *S. mutans* [46]. Oktay et al. discovered that *Candida* biofilm formation was reduced on smooth surfaces in an in vitro study [47]. Therefore, polishing procedures may cut down resin matrix exposure and be beneficial to decrease biofilm formation on the surface of RBC restorations. Our study's results support the prior observations of higher dental plaque formation on rough surfaces and, subsequently, biofilm metabolic activity. However, another study regarding biofilm formation and RBCs' finishing and polishing stated that these procedures may unexpectedly play a minor role in bacterial colonization and biofilm formation [48]. Generally, different systems and methods of polishing, RBC selection, and the amount of residual monomer may affect microbial biofilm formation. It should also be noted that ethanol disinfection may have altered the surface properties of RBCs by removing unpolymerized monomers, potentially affecting roughness and biofilm adhesion. While essential for microbial sanitization, this process does not fully replicate clinical conditions, where RBCs undergo natural aging in saliva. Although no surface characterization was performed postdisinfection, future studies should assess ethanol's impact on surface roughness and biofilm formation. However, since all RBC groups underwent the same treatment, any potential effects remained consistent across samples. In the current investigation, we did not discover any significant differences between the growth of *C. dubliniensis* and *C. albicans* on composite discs in laboratory conditions. Nevertheless, *S. sobrinus* grew and formed biofilms faster on both polished and unpolished composites, which were higher than the other studied bacteria. *S. salivarius* demonstrated the least ability. Our result was in agreement with Pitta et al.'s study, which declared that *S. sobrinus* and *S. mutans* revealed higher biofilm formation while *S. salivarius* had the lowest ability for biofilm formation on implant surfaces [49]. Even so, in Lim et al. investigations into the adhesion of cariogenic *streptococci* to orthodontic brackets*, S. mutans* showed the highest amount of adhesion compared to *S. sobrinus* [50]. *S. mutans* possesses specialized adhesion molecules that enable it to adhere strongly to the tooth surface and form stable biofilms. Additionally, it produces an array of extracellular polysaccharides that contribute to the structural integrity of the biofilm and protect the bacteria from the host's immune response. On the other hand, *S. sobrinus* exhibits a higher acidogenic potential, producing more lactic acid, which further contributes to enamel demineralization and caries progression [51]. *S. salivarius*, in contrast, has been associated with a protective role against caries, as it produces bacteriocins and hydrogen peroxide, which inhibit the growth of cariogenic species [52]. These differences may also have a role in the biofilm formation in our study, as they also can affect targeted preventive strategies and therapeutic interventions to manage dental biofilm and caries disease effectively [53]. These results revealed the importance of material selection and polishing procedures in minimizing biofilm formation on RBCs. Moreover, the significant reduction in biofilm adhesion on polished surface RBCs highlights the need for thorough finishing and polishing during restorations. Clinicians should study biofilm resistance together with mechanical properties when selecting RBCs, mainly in high-plaque areas. Future RBC formulations should purpose to reduce resin matrix exposure and enhance filler surface treatments to further inhibit microbial colonization and enhance patient outcomes.

### 4.1. Future Directions

This study highlights the influence of filler content and surface roughness on biofilm formation, but future research should explore the roles of resin composition and silane coupling agents in microbial adhesion and hydrolytic degradation. Understanding these factors could optimize RBC formulations for reduced biofilm formation and improved clinical performance. Additionally, future studies should investigate the effects of matrix composition, including different monomers and their ratios, on biofilm formation and material degradation. The current study has limitations, such as its in vitro design and use of single-species biofilm models, which do not fully replicate the complexity of oral biofilms. Oral biofilms involve multispecies interactions, salivary pellicles, and host factors, which were not accounted for here. To better evaluate biofilm formation, future research should employ diverse methods, such as CFU counting, confocal microscopy, and pH measurements, alongside the XTT assay, to reduce interspecies variability and provide a more comprehensive understanding of RBC performance. Finally, expanding microbial diversity to include more oral-relevant species (e.g., *Lactobacillus*, *Actinomyces*, and additional *Candida* species) and using multispecies biofilm models would better simulate the oral environment and improve the clinical relevance of findings.

## 5. Conclusion

This study elucidates the significant influence of filler content and surface polishing on the biofilm formation of *Candida* and *Streptococcus* species on RBCs. The findings indicate that the Z250 composite, characterized by its high filler content (68%), exhibited the most substantial biofilm metabolic activity, suggesting a direct correlation between filler amount and microbial colonization. Conversely, the polished specimens of P60 demonstrated a marked reduction in biofilm metabolic activity compared to their unpolished counterparts, highlighting the critical role of surface smoothness in mitigating microbial adherence. In conclusion, optimizing the formulation of RBCs by minimizing excess resin matrix and enhancing surface smoothness can effectively reduce biofilm metabolic activity and consequently lower the risk of secondary caries. These insights are essential for guiding future developments in dental materials aimed at improving oral health outcomes and patient well-being. Further research is warranted to explore the long-term implications of these findings in clinical settings and to refine composite formulations for enhanced performance against microbial colonization.

## Figures and Tables

**Table 1:** Composition of the resin-based composites used in the present study.

Name	Matrix composition	Filler composition and size	Filler volume (%)	Flexural strength (MPa)	Hardness (VHN)
P60	Bis-GMA, UDMA, Bis-EMA	Silica/zirconia, PSD: 0.01–3.5 μm, PSD average: 0.6 μm	61	156	71.4
Z100	Bis-GMA, TEGDMA	Silica/zirconia, PSD: 0.01–3.3 μm, PSD average: 0.6 μm	66	114	61.4
Z250	Bis-GMA, UDMA, Bis-EMA	Silica/zirconia, PSD: 0.01–3.5 μm, PSD average: 0.6 μm	68	138.2	77.5
Z350	Bis-GMA, UDMA, TEGDMA, Bis-EMA	Combination of nonagglomerated/nonaggregated 20-nm silica filler, nonagglomerated/nonaggregated 4–11-nm zirconia filler, and aggregated zirconia/silica cluster filler with a PSD average: 0.6–10 μm	55.6	127.5	69.7

*Note:* Bis-EMA; 2, 2-bis (4-(2-methacryl-oxyethoxy) phenyl) propane, Bis-GMA: bisphenyl A glycidyl methacrylate; TEGDMA: triethyleneglycol dimethacrylate; UDMA: urethane dimethacrylate.

Abbreviations: PSD = particle size distribution and VHN = Vickers hardness number.

**Table 2:** *Candida* spp. biofilm formation absorbance at 570 nm and percent vitality values for different types of resins.

RBC type (number)		*C. albicans* (ATCC 35668)		*C. dubliniensis* (ATCC 35668)		*C. albicans* (clinical)	
		OD value (mean ± SD)	Vitality (%) (mean ± SD)	OD value (mean ± SD)	Vitality (%) (mean ± SD)	OD value (mean ± SD)	Vitality (%) (mean ± SD)
P60	Unpolished (9)	0.12 ± 0.01	42 ± 3.2	0.09 ± 0.01	45.6 ± 3.2	0.15 ± 0.02	70.6 ± 3.2
	Polished (9)	0.085 ± 0.004	28 ± 2.6	0.075 ± 0.012	38 ± 4.5	0.2 ± 0.12	63 ± 2.5

Z100	Unpolished (9)	0.26 ± 0.02	74.1 ± 4.5	0.15 ± 0.04	75 ± 4.8	0.21 ± 0.02	57 ± 1.8
	Polished (9)	0.2 ± 0.036	57 ± 3.8	0.1 ± 0.046	63 ± 2.6	0.1 ± 0.16	39 ± 2.1

Z350	Unpolished (9)	0.24 ± 0.06	79.2 ± 5.1	0.19 ± 0.03	72 ± 3.6	0.3 ± 0.03	78 ± 1.6
	Polished (9)	0.115 ± 0.04	38 ± 2.6	0.094 ± 0.033	53 ± 5.1	0.2 ± 0.23	55 ± 6.1

Z250	Unpolished (9)	0.26 ± 0.03	81.9 ± 4.6	0.2 ± 0.02	87 ± 4.3	0.28 ± 0.02	80 ± 5.3
	Polished (9)	0.2 ± 0.015	63 ± 3.2	0.15 ± 0.042	75 ± 5.6	0.18 ± 0.12	43 ± 4.1

**Table 3:** Results of two-way analysis of variance on *Candida* species.

Independent variables (source of variation)	*C. albicans* (ATCC 35668)			*C. dubliniensis* (ATCC 35668)		
	*F* value	*p* value	Adjusted *R* square	*F* value	*p* value	Adjusted *R* square
RBC type	265.4	< 0.001	0.978	118.8	< 0.001	0.956
Polishing	295.8	< 0.001		181.4	< 0.001	
RBC type and polishing interaction	29.7	< 0.001		19.9	< 0.001	

**Table 4:** *Streptococcus* spp. biofilm formation absorbance at 570 nm and percent vitality values for different types of resins.

RBC type (number)		*S. mutans* (ATCC 35668)		*S. sobrinus* (ATCC 27607)		*S. salivarius* (ATCC 9222)		*S. mutans* (clinical)	
		Mean ± SD	Vitality (%) ± SD	Mean ± SD	Vitality (%) ± SD	Mean ± SD	Vitality (%) ± SD	Mean ± SD	Vitality (%) ± SD
P60	Unpolished (12)	0.1 ± 0.056	50.2 ± 3.8	0.105 ± 0.021	50.0 ± 2.6	0.08 ± 0.014	25.6 ± 2.3	0.16 ± 0.056	77.2 ± 7.8
	Polished (12)	0.06 ± 0.013	24.5 ± 2.3	0.08 ± 0.01	39.3 ± 4.1	0.05 ± 0.01	16.3 ± 1.1	0.07 ± 0.013	34.5 ± 5.3

Z100	Unpolished (12)	0.15 ± 0.05	60.3 ± 3.6	0.185 ± 0.021	86.2 ± 5.1	0.125 ± 0.21	37.8 ± 3.1	0.18 ± 0.05	85.3 ± 6.6
	Polished (12)	0.08 ± 0.14	32.4 ± 2.8	0.09 ± 0.02	43.1 ± 2.9	0.1 ± 0.04	31.0 ± 2.3	0.09 ± 0.14	48.4 ± 3.8

Z350	Unpolished (12)	0.1 ± 0.007	51.0 ± 2.9	0.16 ± 0.09	77.3 ± 4.9	0.11 ± 0.14	34.2 ± 2.9	0.2 ± 0.007	71.0 ± 6.9
	Polished (12)	0.07 ± 0.012	28.3 ± 2.5	0.07 ± 0.01	34.5 ± 2.6	0.06 ± 0.02	19.5 ± 2.7	01 ± 0.012	49.3 ± 4.5

Z250	Unpolished (12)	0.19 ± 0.02	84.3 ± 9.1	0.185 ± 0.007	86.4 ± 6.5	0.23 ± 0.02	70.6 ± 3.6	0.22 ± 0.02	85.3 ± 8.1
	Polished (12)	0.15 ± 0.07	76.1 ± 3.6	0.16 ± 0.09	77.6 ± 5.1	0.16 ± 0.05	49.6 ± 3.1	0.13 ± 0.07	41.1 ± 2.6

**Table 5:** Results of two-way analysis of variance on *Streptococcus* species.

Independent variables (source of variation)	*S. mutans* (ATCC 35668)			*S. sobrinus* (ATCC 27607)			*S. salivarius* (ATCC 9222)		
	*F* value	*p* value	Adjusted *R* square	*F* value	*p* value	Adjusted *R* square	*F* value	*p* value	Adjusted *R* square
RBC type	1.18	0.384	0.000	183.2	< 0.001	0.981	240.2	< 0.001	0.978
Polishing	0.095	0.762		451.5	< 0.001		132.4	< 0.001	
RBC type and polishing interaction	1.07	0.388		74.3	< 0.001		11.5	< 0.001	

## Data Availability

The data used to support the findings of this study were supplied by Shiraz University of Medical Sciences. Requests for access to these data should be made to Kamiar Zomorodian, zomorodian@sums.ac.ir or kzomorodian@gmail.com.
